# Transcriptomic and proteomic profiling reveal immune and metabolic dysregulation in the colonic mucosa of people living with HIV with incomplete immune recovery

**DOI:** 10.3389/fimmu.2025.1635523

**Published:** 2025-09-17

**Authors:** Mari Kaarbø, Mingyi Yang, Malin Holm Meyer-Myklestad, Arvind Y. M. Sundaram, Mirta Mittelstedt Leal de Sousa, Animesh Sharma, Asle W. Medhus, Anne Margarita Dyrhol-Riise, Dag Kvale, Johannes R. Hov, Pål Aukrust, Magnar Bjørås, Dag Henrik Reikvam

**Affiliations:** ^1^ Department of Microbiology, Oslo University Hospital, Oslo, Norway; ^2^ Department of Infectious Diseases, Oslo University Hospital, Oslo, Norway; ^3^ Department of Medical Genetics, Oslo University Hospital and University of Oslo, Oslo, Norway; ^4^ Department of Clinical and Molecular Medicine, Norwegian University of Science and Technology, Trondheim, Norway; ^5^ Proteomics and Modomics Experimental Core Facility at Norwegian University of Science and Technology, and the Central Norway Regional Health Authority, Trondheim, Norway; ^6^ Department of Gastroenterology, Oslo University Hospital, Oslo, Norway; ^7^ Institute for Clinical Medicine, University of Oslo, Oslo, Norway; ^8^ Norwegian PSC Research Centre and Section of Gastroenterology, Department of Transplantation Medicine, Oslo University Hospital, Oslo, Norway; ^9^ Research Institute of Internal Medicine, Division of Surgery, Inflammatory Diseases and Transplantation, Oslo University Hospital, Oslo, Norway; ^10^ Section of Clinical Immunology and Infectious Diseases, Oslo University Hospital, Oslo, Norway; ^11^ Centre for Embryology and Healthy Development, University of Oslo and Oslo University Hospital, Oslo, Norway

**Keywords:** immunological non-responders, mucosal immunology, inflammatory bowel disease, metabolic, gut mucosa, lamina propria, RNA-seq, shotgun mass spectrometry

## Abstract

**Background:**

People living with HIV are called immunological non-responders (INR) when their CD4+ T cell count is not restored to immunocompetent levels, despite successful viral suppression. INR have increased risk of progression to AIDS, non-AIDS related morbidity, and death. Impaired mucosal barrier function is a prevailing hypothesis for why INR among people with HIV (PWH) have persistently low CD4+ T cell counts.

**Objective:**

To understand the molecular mechanisms behind incomplete immune recovery in INR, we analyzed gene regulation and protein expression in gut tissues from INR, immunological responders (IR) and healthy controls (HC).

**Methods:**

The transcriptome was assessed by RNA-sequencing (RNA-seq) and the proteome was examined using shotgun proteomic mass spectrometry in mucosal biopsies from the sigmoid colon and terminal ileum.

**Results:**

In INR compared to IR, we identified 3326 differentially expressed genes (DEGs) in the colon while no DEGs were observed in the ileum. Gene ontology (GO) analyses revealed that the DEGs in colon of INR, compared to IR, predominantly involved pathways related to immune response, metabolism, and cellular processes. Notably, GO analysis highlighted downregulation of genes associated with B cell-mediated immunity and adaptive responses in INR. Deconvolution analysis indicated that these transcriptomic changes were not solely due to shifts in immune cell composition. Proteomic analysis supported these findings, showing more differential protein composition between INR and IR in colon than ileum. These proteins are associated with the regulation of adaptive immune signaling and essential cellular processes, including cell signaling, tissue repair, and growth, all of which are characteristic features of inflammatory bowel disease (IBD).

**Conclusion:**

Our findings suggest that incomplete immune recovery during anti-retroviral therapy in PWH is associated with specific dysregulations in the molecular environment of the sigmoid colon, which may share mechanisms with IBD. The identified macromolecules may serve as potential targets for adjuvant treatment to improve the prognosis for INR.

## 1 Introduction

Human immunodeficiency virus (HIV) infection affects more than 40 million people worldwide (1). During the last decades, the introduction of antiretroviral treatment (ART) has dramatically improved the prognosis for people with HIV (PWH). ART suppresses viral replication efficiently and allows the immune system to recover over time for the majority of PWH. However, in 10 to 15% of PWH, efficient treatment with persistent viral suppression fails to restore the level of CD4^+^ T cells (2, 3). This group is termed immunological non-responders (INR), and their impaired immunological response is associated with an increased risk of progression to AIDS and non-AIDS related morbidity and mortality (4–7). INR also show signs of increased and persistent inflammation and immune activation compared to immune responders (IR) who have normal CD4^+^ T cell counts. The molecular causes underlying the INR phenotype remain elusive (8–12).

The gastrointestinal (GI) tract is the largest lymphoid organ of the human body and plays an important role in HIV persistence and viral replication (13, 14). During acute HIV infection, the gut suffers a dramatic destruction of mucosal CD4^+^ T cells, including the T helper (Th)17 and Th22 subsets, which both are essential to support the gut epithelium (13). As a result, untreated HIV infection leads to disruption of the gut mucosal barrier allowing translocation of luminal microbial products that subsequently promote immune activation and inflammation not only in the GI tract but also systemically (13). Thus, gut mucosal barrier dysfunction may contribute to inadequate immunological response to ART and the INR phenotype (15).

We have shown that INR have signs of increased enterocyte damage and mucosal immune dysfunction in gut biopsies as compared to IR (16, 17). Importantly, in gut mucosal biopsies, INR had a lower fraction of CD4+ T cells and a higher fraction of Th22 cells compared to IR, which were restricted to the sigmoid colon (colon) compared with terminal ileum (ileum) (16). Additionally, levels of the enterocyte damage marker intestinal fatty acid binding protein were elevated in INR compared to IR. Furthermore, regenerating islet-derived protein 3 alpha (REG3α), a novel marker of enterocyte damage, also showed a tendency to be higher in INR than in IR (16). To gain a deeper insight into mucosal dysregulation in INR and to identify potential molecular targets for adjuvant treatment, we performed untargeted transcriptome and proteome analyses in the sigmoid colon and terminal ileum of INR, IR, and healthy control (HC) groups in this study.

## 2 Materials and methods

### 2.1 Participant and study design

The participants and study design have previously been presented (16). PWH were recruited from the outpatient clinic of the Department of Infectious Diseases, Oslo University Hospital. INR were defined as < 400 CD4^+^ T cells/µL and IR as having > 600 CD4^+^ T cells/ µL for longer than 3.5 years. All INR (n=4 for RNA-seq and n=5 for proteomics) and IR (n=5, RNA-seq and proteomics) included were male, on continuous efficient ART for > 4 years, < 50 HIV RNA copies/ml plasma > 3.5 years, and the groups were matched on nadir CD4 count (before initiating ART) and age. Men without HIV referred to colonoscopy for control after polyp removal and age-matched with INR and IR, were enrolled as healthy controls (HC, n=5 for RNA-seq and proteomics). The characteristics of the study group are presented in **Table 1**.

**Table 1:** Characteristics of study participants used for transcriptomic (RNA-sequencing) (A) and proteomic (mass spec) (B) studies.

A	INR (n=4)	IR (n=5)	HC (n=5)	P-value
Age, y, median	54.4 (43.7-64.3)	52.7 (50.0-62.5)	54.9 (51.1-60.5)	0.99^1)^
Nadir CD4+ cell count, cells/µl, median	35 (10-139)	73 (13-195)	N/A	0.39^2)^
CD4 + T cell count at enrolment, cells/µl, median	244 (180-311)	739 (474-797)	N/A	0.02^2)^
Time since HIV seroconversion, y, median	9.36 (7.69-10.4)	25 (15.3-31.3)	N/A	0.11^2)^

B	INR (n=5)	IR (n=5)	HC (n=5)	P-value
Age, y, median	47.8 (46.5-55.0)	50.4 (47.1-62.3)	50.2 (40.5-53.9)	0.72^1)^
Nadir CD4+ cell count, cells/µl, median	156 (80-184)	108 (20-166)	N/A	0.51^2)^
CD4 + T cell count at enrolment, cells/µl, median	363 (331-389)	1009 (833-1075)	N/A	0.008^2)^
Time since HIV seroconversion, y, median	22.1 (7.36-23.9)	16.2 (11.3-23)	N/A	>0.99^2)^

^1)^ Kruskal-Wallis.

^2)^ Mann-Whitney INR versus IR.

### 2.2 Sample collection

Single pinch biopsies were obtained from the terminal ileum and the sigmoid colon by colonoscopy as previously described (16). The biopsies were either preserved in RNAlater (Thermo Fisher Scientific, Waltham, MA, USA) at -20°C for transcriptome analyses (RNA-sequencing) or snap frozen in 1.5 mL tubes in liquid nitrogen and stored at -80°C for proteome analyses by liquid chromatography–mass spectrometry (LS-MS/MS).

### 2.3 RNA-sequencing (RNA-seq)

#### 2.3.1 Sample preparation for RNA-seq

Total RNA was isolated from biopsies stored in RNA later using Allprep DNA/RNA Mini kit (Qiagen, Hilden, Germany) according to the manufacturer’s instructions with a few modifications as described in (18). The samples were lysed in RLT buffer with β-mercaptoethanol and homogenized using 0.1 mm zirconia steel beads (BioSpec Products, Bartlesville, OK, USA) and 5 mm stainless steel beads (Qiagen, Hilden, Germany) in a MiniBeadBeater (BioSpec Products, Bartlesville, OK). The RNA was treated with DNAse on the column for 15 minutes and eluted in molecular grade H_2_0 and stored at - 80°C. The quantity and quality were measured using Nanodrop (Thermo Fisher Scientific, Waltham, MA) and Tapestation (Agilent Technologies, Santa Clara, CA), respectively.

#### 2.3.2 RNA-seq

Total RNA samples (RIN > 6.3) were used for directional library preparation (Illumina mRNA stranded prep kit, San Diego, CA) using polyA selection and were sequenced on five lanes in Illumina HiSeq, 150 bp paired end (Illumina, San Diego, CA). Additional details can be found in the supplementary methods section. A table of differentially expressed genes (DEGs) is available in **Supplementary Table S1**.

#### 2.3.3 Deconvolution analyses for estimating immune cell proportions from RNA-seq data

To estimate the proportions of immune cell subsets from RNA-seq data, we conducted deconvolution analysis using the R package “granulator” (19). Within this package, we employed the quadratic programming with non-negative least-squares constraints linear model (qprogwc), as described in (19, 20). We utilized the “sigMatrix_ABIS_S0” reference dataset, which contains transcription levels of 1,200 genes across 17 immune cell types derived from single-cell RNA-seq data, to accurately represent the various immune cell populations. The normalized mRNA counts (TPM, transcripts per million) from our RNA-seq dataset served as input for predicting the proportions of different cell subsets.

#### 2.3.4 Gene set enrichment analysis on gene ontology (GSEA on GO) terms

GSEA was performed to explore the enrichment in gene ontology (GO) terms (biological process, molecular function and cellular component) by comparing transcription profile between conditions, using the complete list DEGs as input, ranking them based on expression level and calculation of enrichment score with GSEA tool implemented in R package clusterProfile (21, 22).

### 2.4 Liquid chromatography–tandem mass spectrometry (LC-MS/MS)

#### 2.4.1 Sample preparation

The total proteomes of biopsies (n=5 per group) from sigmoid colon and terminal ileum were analyzed. Total protein was isolated from snap-frozen biopsies using Allprep DNA, RNA and protein kit (Qiagen, Hilden, Germany) according to the manufacturer’s instructions with a few modifications: the samples were lysed in RLT buffer with β-mercaptoethanol and homogenized using a QIAshredder (Qiagen, Hilden, Germany). The protein fraction was precipitated, washed in 70% ethanol, dried and stored at -80°C. The peptide concentration was measured in a NanoDrop One C (Thermo Fisher Scientific, Waltham, MA) at 205 nm, Scopes’ method (23). For detailed preparation method for LC-MS/MS, please refer to the **Supplementary Material and Methods**.

#### 2.4.2 Shotgun proteomic analysis

Protein analyses were performed on a LC-MS/MS platform consisting of an EASY-nLC 1200 UHPLC system coupled to a Q Exactive HF mass spectrometer operating in FullMS-ddMS2 mode (Thermo Fisher Scientific, Waltham, MA). Protein concentrations were quantified by processing MS data using MaxQuant (MQ) v.1.6.6.0 (24). The protein data were further analyzed using an R DEP package (Differential Enrichment analysis of Proteomics data, Version 1.12.0) by which the DEPs were identified using the protein-wise linear model (limma package inside DEP) combined with empirical Bayes statistics. DEPs were defined by adj p-value < 0.05, according to the Benjamini-Hochberg Method (25). GO and Kyoto Encyclopedia of Genes and Genomes (KEGG) pathway enrichment analyses were performed using R package clusterProfile (3.18.1). The data were visualized using R packages including ggplot2 and VennDetail. The detailed method is included in **Supplementary Material** and **Material**. The mass spectrometry proteomics data have been deposited to the ProteomeXchange Consortium via the PRIDE (26) partner repository with the dataset identifier PXD041255. A table of differentially expressed proteins (DEPs) is available in **Supplementary Table S2**.

ImmuneSigDB (Immune Signatures Database) was used in conjunction with the GSEA (Gene Set Enrichment Analysis) software to map the differentially expressed proteins in sigmoid colon.

### 2.5 Integrative transcriptomic and proteomic analysis

To integrate the transcriptomic and proteomic datasets from colonic biopsies, we performed a multi-step correlation analysis to identify and characterize concordant molecular features between immunological non-responders (INR) and immunological responders (IR). We compared the DEGs and DEPs lists to identify genes that were significantly dysregulated in both the transcript and protein levels. We then constructed a gene–protein correlation network using the overlapping genes. In this network, node sizes reflected the product of the absolute log_2_ fold changes from RNA and protein data, while edge weights indicated the strength of correlation between RNA and protein expression levels. To quantify these relationships, we performed correlation analysis using scatterplots of log_2_ fold change values for each overlapping gene and calculated Pearson correlation coefficients (by R built-in function “cor”) to assess the degree of concordance. To create a network plot, the correlation matrix was filtered by coefficient R > 0.5, then further converted into graph matrix by R package igraph using function “graph_from_adjacency_matrix” (parameter weighted = True, mode = “undirected”), further converted as network object by R package Intergraph. The network plot was created from network object using ggplot2 package. Functional annotation of the overlapping genes was carried out using public gene annotation databases, including UniProt, Gene Ontology (GO), and GeneCards, with an emphasis on immune signaling, translational regulation, and mucosal barrier function (by R package clusterProfile and AnnotationDbi). Finally, we performed unsupervised hierarchical clustering based on the integrated RNA and protein expression levels of the overlapping genes (R package p heatmap). Clustering was done using Euclidean distance and complete linkage to assess whether these features could stratify INR and IR samples.

### 2.6 Statistical analyses

A modified t-test (Wald test) was used in statistical p value analysis for the RNA-seq data. DEGs were defined as adjusted p-value < 0.05. Analyses of DEPs for the proteomics data were performed using Empirical Bayes statistics test. For both RNA-seq and proteomics analyses, the adjusted p-value was used to decrease the false discovery rate (25). The Chi-square test was used to compare regulation in sigmoid colon and terminal ileum (27). For comparisons between paired groups (e.g., INR versus IR within subjects), paired two-tailed t-test was used, while one-way ANOVA was applied to assess differences across more than two paired groups for the deconvolution assay. Gene Ontology (GO) term enrichment was carried out with clusterProfiler, using FDR (adjusted p-value). The correlation between RNA and protein expression levels was assessed by Pearson correlation coefficient. A p-value < 0.05 or adjusted p-value < 0.05 was considered statistically significant throughout.

## 3 Results

### 3.1 INR exhibit distinct RNA regulation compared to IR exclusively in the sigmoid colon

**Figure 1:**
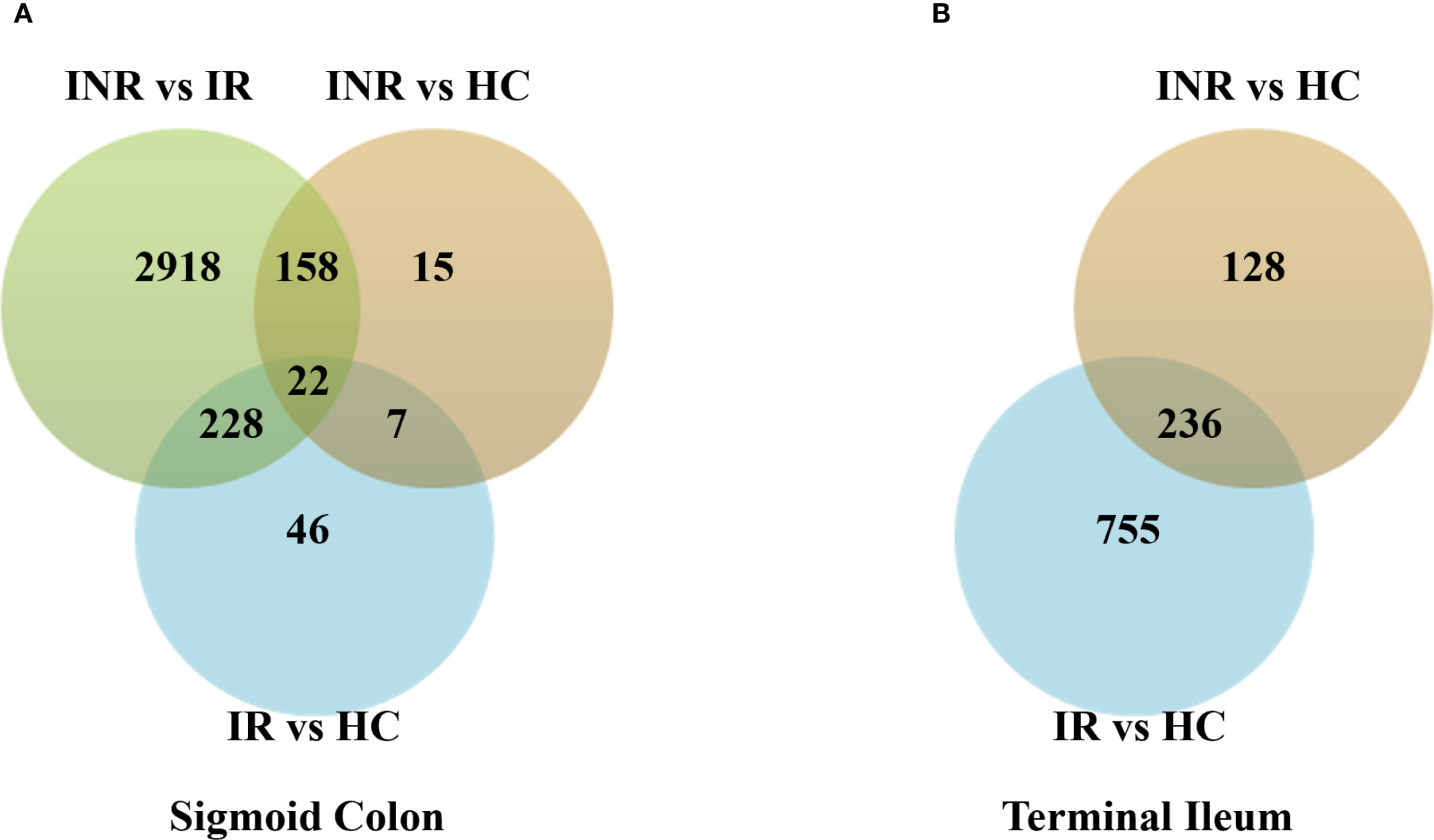
Transcriptional regulation in immunological non-responders, immunological responders and healthy controls in **(A)** sigmoid colon and **(B)** terminal ileum. Venn diagrams illustrating numbers of uniquely and commonly differentially expressed transcripts (p adj < 0.05) in INR (n=4), IR (n=5) and HC (n=5). Total RNA was isolated from gut mucosal endoscopic pinch biopsies and transcriptomic analyses were performed by RNA-sequencing. Modified t-test (Wald test) was used to identify differential expression (p adj < 0.05). The Chi-square test was used to calculate the difference in expression between the two tissues (p < 0.0001). INR, immunological non-responders; IR, immunological responders; HC, healthy controls.

RNA-seq was performed on endoscopic pinch biopsies of gut mucosa from INR, IR and HC to examine transcriptome regulation in the sigmoid colon and terminal ileum. The participant characteristics are outlined in **Table 1**. Differential gene expression analysis, performed using negative binomial GLM fitting and Wald tests, revealed significantly higher expression differences in the sigmoid colon compared to the terminal ileum (p < 0.0001) (**Figures 1A, B**). In the colon, we identified 3326 DEGs when comparing INR to IR, and 202 DEGs when comparing INR to HC (**Figure 1A**). Strikingly, although both INR and IR showed several differences compared with HC (364 and 991 DEGs, respectively) in the TI, there was no difference in the transcriptome between INR and IR in this compartment (**Figure 1B**).

#### 3.1.1 Dysregulation of transcripts related to metabolism, immune response, and stimulus reactivity in the sigmoid colon of INR

**Figure 2:**
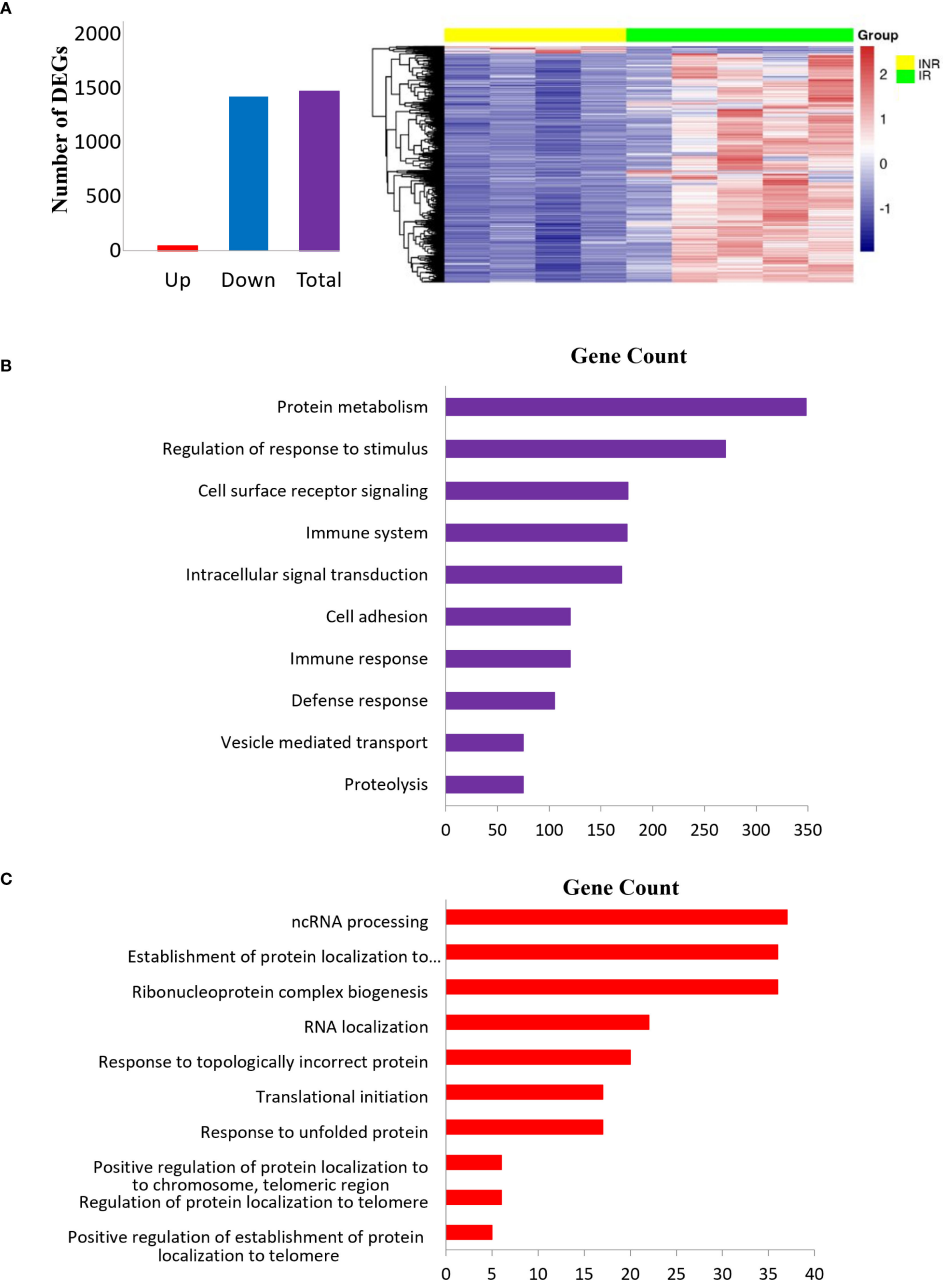
Differentially expressed transcripts in INR compared to IR in sigmoid colon. **(A)** Bar plot (left) and heatmap (right) showing top DEGs with fold change >2 and adjusted p < 0.05 in INR versus IR in the colon. The heatmap displays Z-scores, where positive values indicate expression above the mean across all samples, and negative values indicate expression below the mean. **(B)** Gene ontology enrichment analyses of DEGs with two-fold or greater regulation in INR versus IR in the colon. The pathways (y-axis) are ranked by enrichment (x-axis); **(C)** Gene ontology analysis of upregulated DEGs in INR versus IR in the colon. The canonical pathways (y-axis) are ranked by enrichment (x-axis). Modified t-test (Wald test) was used to identify differential expression (p adj < 0.05). INR, immunological non-responders; IR, immunological responders; HC, healthy controls; DEGs, Differentially expressed genes.

In sigmoid colon, 2579 mRNAs were *downregulated* in INR compared to IR while 747 were *upregulated*. Among these 3326 DEGs, 1471 DEGs had two-fold change or more (p adj < 0.05) (**Figure 2A**). GO analyses of DEGs with two-fold change or more in INR versus IR showed that these transcripts were implicated in critical processes such as protein metabolism, regulation of stimulus and immune responses signal transduction, defense responses, and cell adhesion (**Figure 2B**), GO analyses of the 747 mRNAs that were significantly *upregulated* in INR compared to IR (p adj < 0.05, **Figure 2C**) indicated involvement in cellular processes such as regulation of RNA (ncRNA, RNA metabolism and localization) and protein regulation (localization to organelle and telomere and protein folding).

Several of the mRNAs significantly dysregulated in the colonic tissue of INR (adjusted p < 0.05) have been previously implicated in HIV pathogenesis and are linked to diverse biological pathways. HIV entry was associated with *cluster of differentiation 4* (CD4, log_2_fold -0.678), the primary receptor for viral attachment and internalization (2, 3, 28, 29). Immune response modulation was linked to multiple molecules, including *Chemokine (C-X-C motif) ligand 1* (CXCL1, also known as GRO-α, log_2_fold 1.225), which has been shown to enhance HIV replication (30–32); *Interleukin-21 Receptor* (IL21R, log_2_fold -1.899), important for T cell and B cell function (33); *Transforming Growth Factor Beta 1* (TGFB1, log_2_fold -1.028), known to regulate immune activation (34); and *Human Leukocyte Antigen E* (HLA-E, log_2_fold -0.594), involved in immune evasion and NK cell regulation (35, 36). T cell activation and function were associated with *Tyrosine Phosphatase Non-Receptor Type 22* (PTPN22, log_2_fold 0.697), which modulates T cell receptor (TCR) signaling (37), and *Granzyme A* (GZMA, log_2_fold 0.764), a key effector in cytotoxic lymphocytes (38). Immune signaling pathways included *Mitogen-Activated Protein Kinase Kinase Kinase 5* (MAP3K5, log_2_fold 0.521), also known as ASK1, which regulates stress-induced apoptosis and interacts with multiple HIV proteins (39–41). Lastly, MHC class II regulation was linked to the *Class II Major Histocompatibility Complex Transactivator* (CIITA, log_2_fold -1.138), a master regulator of antigen presentation, which also plays a complex role in HIV pathogenesis (42–44).

When comparing INR to both IR and HC, the majority of the common transcripts were *downregulated* in INR (**Supplementary Figure S1A**) and typically involved in protein transport, metabolism and cellular localization (**Supplementary Figure S1B**).

#### 3.1.2 Downregulation of transcripts associated with B cell-mediated immunity in the sigmoid colon of INR

**Figure 3:**
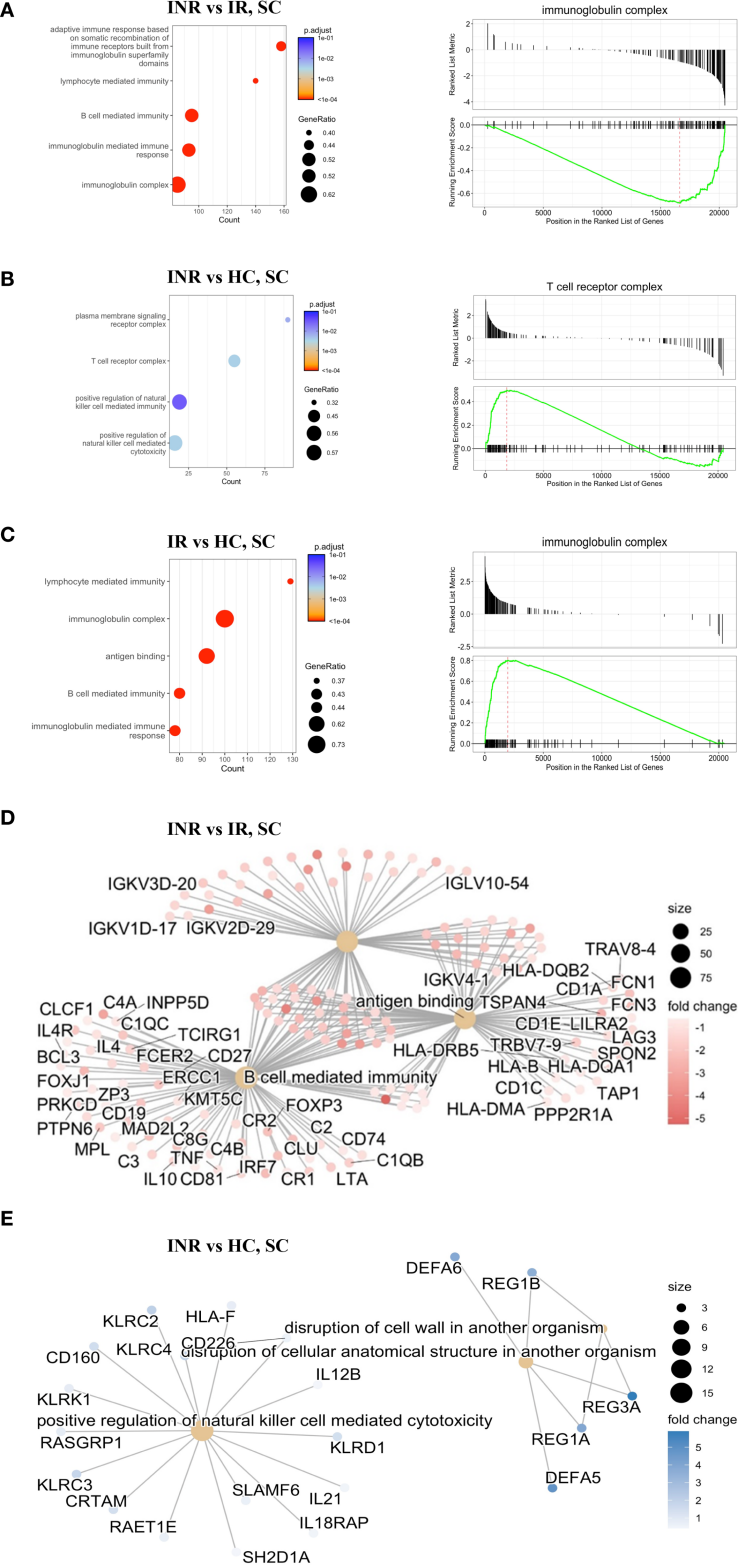
Gene Set Enrichment Analysis (GSEA) by Gene Ontology from RNA-seq data **(A)** Top five enriched GO terms (bar plot, left) comparing colon samples from INR versus IR. A representative enrichment plot (right) shows the running enrichment score and position of genes in the preranked list. **(B)** Top five enriched GO terms (bar plot, left) comparing INR versus HC in the colon, with a representative enrichment plot shown on the right. **(C)** Top five enriched GO terms (bar plot, left) comparing IR versus HC in the colon, with a representative enrichment plot shown on the right. **(D)** Gene network illustrating the top three enriched GO terms in colon samples from immune non-responders (INR) versus immune responders (IR). **(E)** Gene network showing the top three enriched GO terms in colon samples from INR versus healthy controls. INR, immunological non-responders; IR, immunological responders; HC, healthy controls; GSEA, Gene Set Enrichment Analysis; GO, Gene Ontology; RNA-seq, RNA sequencing; SC, sigmoid colon.

To further investigate the immune pathways underlying the dysregulation observed in colon of INR, we performed Gene Set Enrichment Analysis (GSEA) using the Gene Ontology (GO) database, ranking all genes based on transcript levels derived from RNA-seq data (**Figure 3**). When comparing the colon of INR with IR, there was significant downregulation in several key immune processes related to the adaptive immune response (**Figures 3A, D**). Specifically, we observed reduced expression in pathways involved in B-cell mediated immunity, antigen binding, and the immunoglobulin mediated immune response. These findings are visually summarized in **Figure 3D**, highlighting the downregulated factors in INR compared to IR including several transcripts related Ig components (e.g. Immunoglobulin Kappa Variable Cluster, IKGVs) and B cell stimulating cytokines (e.g. interleukin (IL)-4 and IL-10). In contrast, pathways involved in the adaptive immune response were upregulated in IR compared to HC (**Figure 3C**). Interestingly, genes within the immunoglobulin complex showed a positive enrichment score (upregulated) in IR compared to HC but were downregulated in INR compared to IR (**Figure 3A**). When analyzing the comparison between INR and HC, no significant changes in this gene ontology (GO) term were found (**Figure 3B**), suggesting that immunoglobulin complex genes are specifically upregulated in IR but not in INR relative to healthy controls.

#### 3.1.3 Upregulation of transcripts associated with NK cell-mediated cytotoxicity in the sigmoid colon of INR compared to healthy controls

When comparing colon of INR to colon of HC, transcripts associated with positive regulation of natural killer (NK) cell mediated toxicity and disruption of cellular anatomical structures were upregulated in INR (**Figure 3B**). In addition, there were alterations in the plasma membrane signaling receptor complex and the T-cell receptor complex. The specific factors involved in some of the upregulated processes are represented in **Figure 3E**. Notably, the disruption of the cell wall involving REG3α, among others, which we previously have shown to have a tendency to be higher in INR than in IR (16), was upregulated in INR compared to healthy controls. When comparing INR to both IR and HC, the majority of the common transcripts were downregulated in INR (**Supplementary Figure S1A**), and typically involved in protein transport, metabolism and cellular localization (**Supplementary Figure S1B**).

#### 3.1.4 Deconvolution analysis highlights variations in immune cell distribution among PLW groups and healthy controls

**Figure 4:**
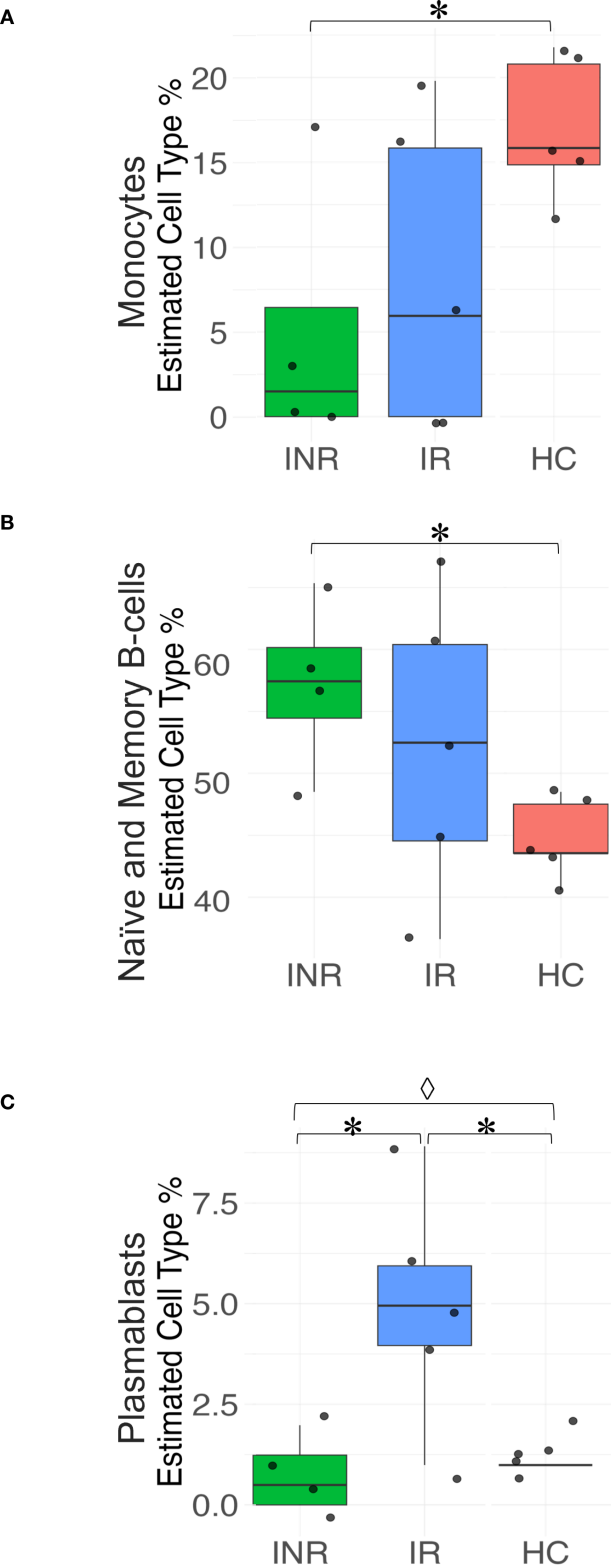
Deconvolution analyses of immune cell subsets in the sigmoid colon. Box plots illustrating the relative abundance of **(A)** Monocytes; **(B)** Naïve and memory B cells; and **(C)** Plasmablasts in sigmoid colon from INR, IR, and HC. Statistical significance is indicated as P<0.05 (*Paired t-test; ◊ one-way ANOVA). Box plots display the median, interquartile range, and minimum/maximum values. INR, immunological non-responders; IR, immunological responders; HC, healthy controls.

To investigate whether the differences observed in transcriptome analyses reflect variations in immune cell distribution among biopsy specimens from the different PLW groups and healthy controls (HC), we conducted a deconvolution analysis of the RNA-seq data (**Figure 4**). The analysis revealed a significant decrease in monocytes in INR compared to HC (Paired t-test, p= 0.049) (**Figure 4A**). Conversely, there was a significant increase in the proportion of memory B cells in INR compared to HC (Paired t-test, p=0.029) (**Figure 4C**). Additionally, our deconvolution analysis indicated a five-fold significant increase in plasmablasts in IR compared to HC (Paired t-test, p =0.030) (**Figure 4C**). However, although the level of plasmablasts was significantly lower in INR compared to those of IR (Paired t-test, p=0.028) and differed significantly between all groups (one tailed ANOVA, p = 0.008) (**Figure 4C**), there were no significant differences in the proportion of naïve and memory B cells between INR and IR (**Figure 4B**). This suggests that the differences in transcriptome analyses indicating reduced B cell mediated immunity in INR as compared with IR (**Figure 3D**) do not merely reflect variations in the distribution of these cells in the biopsy specimens.

### 3.2 Greater protein dysregulation observed in the sigmoid colon of INR compared to IR, in contrast to the terminal ileum

**Figure 5:**
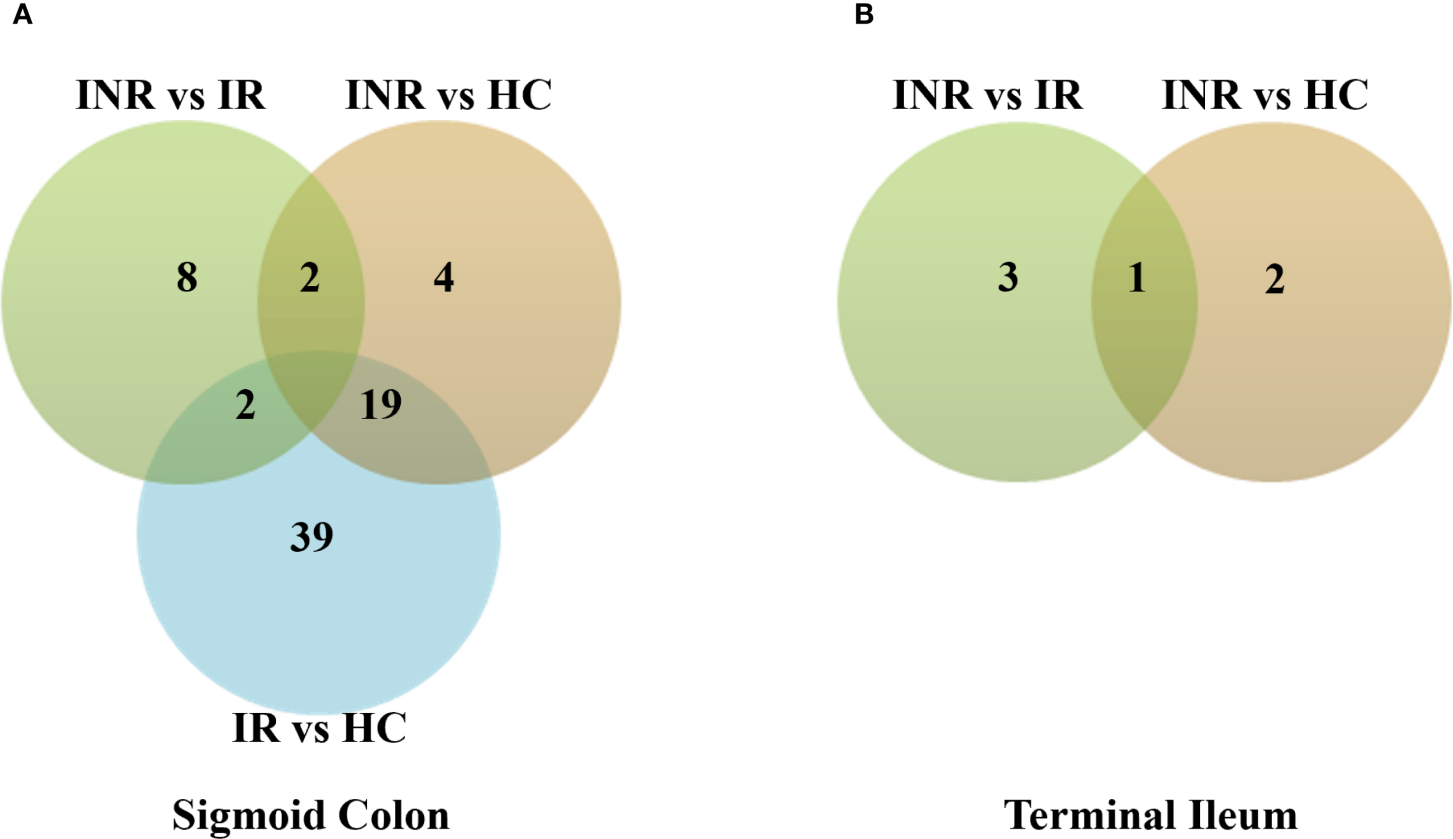
Protein regulation in INR, IR and HC in **(A)** sigmoid colon compared to **(B)** terminal ileum. Venn diagram showing numbers of uniquely and commonly regulated proteins, DEPs (p adj < 0.05). Total protein was isolated from gut mucosal endoscopic pinch biopsies protein levels and analysed by mass spectrometry. Empirical Bayes statistics test was used to identify differential regulation (p adj < 0.05). Chi-square test was used to calculate difference in regulation between the two tissues (p adj < 0.0001). INR, immunological non-responders; IR, immunological responders; HC, healthy controls; DEPs, Differential Enrichment analysis of Proteomics data.

We next examined protein regulation in biopsies from sigmoid colon and terminal ileum by global proteomic analyses, focusing on INR and IR that was the main predefined comparison of the study. We detected a total of 4027 common proteins for all three groups in both tissues after data cleaning and filtering out proteins that were not identified in all replicates for at least one group (INR, IR and/or HC). The data were further evaluated for differential expression by empirical Bayes statistics test. Consistent with our findings at the transcriptional level, more proteins were differentially regulated in sigmoid colon compared to terminal ileum (p < 0.0001; **Figures 5A, B**). In the paired comparisons from the three groups (IR, INR and HC), we identified a total of 74 differentially regulated proteins in sigmoid colon (**Figure 5A**) and six in terminal ileum (**Figure 5B**). Twelve proteins were differentially regulated between INR and IR in sigmoid colon but only four in terminal ileum (**Figures 5A, B**, respectively).

#### 3.2.1 Differential pathway and protein expression analysis in the sigmoid colon of INR versus IR: implications for immune response regulation

**Figure 6:**
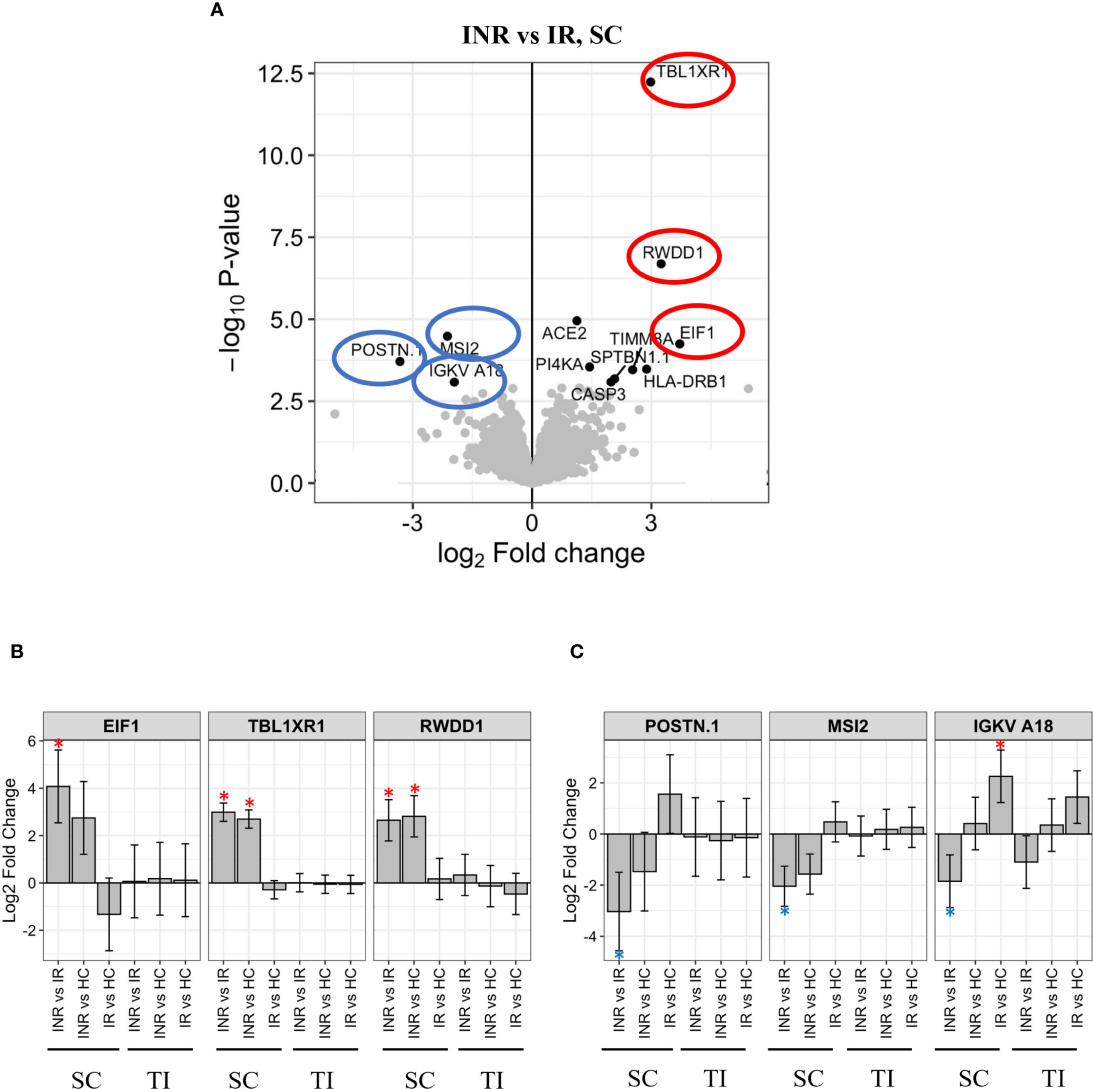
Protein regulation in INR versus IR in sigmoid colon **(A)** Volcano plot illustrating proteins significantly regulated in INR compared to IR in sigmoid colon. Y-axis shows -log10 adjusted p-value, X-axis log2 fold change. Significant DEPs are in black dots with the corresponding name. Grey dots illustrate non-significant DEPs. The most significantly upregulated proteins in INR versus IR (-log10 adjusted p-value > 2.5) are highlighted in red circles, significantly downregulated proteins are highlighted in blue circles. **(B)** Levels of the most upregulated proteins in INR versus IR are shown for all groups in both tissues. The protein intensity (log2) (Y-axis) is shown for each group with significant differences (p < 0.05) marked *. **(C)** Levels of the most downregulated proteins in INR *vs* IR shown for all groups in both tissues. The protein intensity (log2) (Y-axis) is shown for each group, significant differences (p < 0.05) are marked *. Error bars represent 95% confidence intervals. INR, immunological non-responders; IR, immunological responders; HC, healthy controls; DEP, Differential Enrichment analysis of Proteomics data; E1F1, *Eukaryotic translation initiation factor*; IGKV18, *Antibody immunoglobulin kappa variable A18*; MSI2, *Musashi RNA binding protein 2*; POSTN1, *Periostin*; RWDD1, *transcription factor RWD domain containing 1*; SC, sigmoid colon; TBLXR1, *Transducin β-like 1 X-linked receptor 1*; TI, terminal ileum.

Proteomic analysis of the sigmoid colon revealed distinct differences in protein expression between INR and IR, with significant implications for mucosal immune activity. Among the proteins most upregulated in INR samples were *Eukaryotic translation initiation factor 1* (EIF1), *Transcriptional regulator transducin β-like X-linked receptor 1* (TBLXR1), and *Transcription factor RWD domain-containing protein 1* (RWDD1) (**Figures 6A, B**), proteins associated with immune function and carcinogenesis (45–47). In contrast, *Periostin* (POSTN1), *Musashi RNA-binding protein 2* (MSI2), and *Immunoglobulin kappa variable A18* (IGKV18*)* were significantly downregulated in INR (**Figure 6C**). POSTN1 contributes to extracellular matrix remodeling and tissue repair (48, 49). MSI2 regulates RNA metabolism and autophagy, and IGKV18 is associated with B cell–mediated immune responses (50, 51). Additional differentially expressed proteins included *Angiotensin-converting enzyme 2* (ACE2), which was upregulated in INRs and is known for its role in mucosal receptor signaling and as a co-receptor exploited by several viruses. *Caspase-3* (CASP3), a central mediator of apoptosis, was also elevated in INR samples. In relation to antigen presentation, *Major histocompatibility complex, class II, DR beta 1* (HLA-DRB1) was upregulated, consistent with reports linking specific *HLA-DRB1* alleles to variations in HIV-1 susceptibility, disease progression, and viral control (52, 53). Regarding intracellular signaling, *Phosphatidylinositol 4-kinase alpha* (PI4KA) was upregulated, while *MSI2* was downregulated, suggesting alterations in both phosphoinositide signaling and RNA regulation. Structural and mitochondrial components were also affected: *Spectrin beta chain, non-erythrocytic 1* (SPTBN1), which supports cytoskeletal architecture (54), and *Translocase of inner mitochondrial membrane 8A* (TIMM8A), which facilitates mitochondrial protein import (55), were both upregulated in INR samples. Collectively, these expression profiles indicate widespread differences in immune regulation, apoptosis, antigen processing, cellular signaling, and homeostasis between INR and IR at the mucosal level (32, 56). Supporting these findings, ImmuneSigDB analyses of the differentially expressed proteins in the sigmoid colon of INR and IR revealed significant differences in pathways regulating memory B cells, CD4 T-helper cells, and dendritic cells (DCs) between the two groups. GO analyses further revealed significant differences in cytokine production pathways between INR and IR.

#### 3.2.2 Significant downregulation of mitochondrial and cell cycle-related proteins in the terminal ileum of INR compared to IR

**Figure 7:**
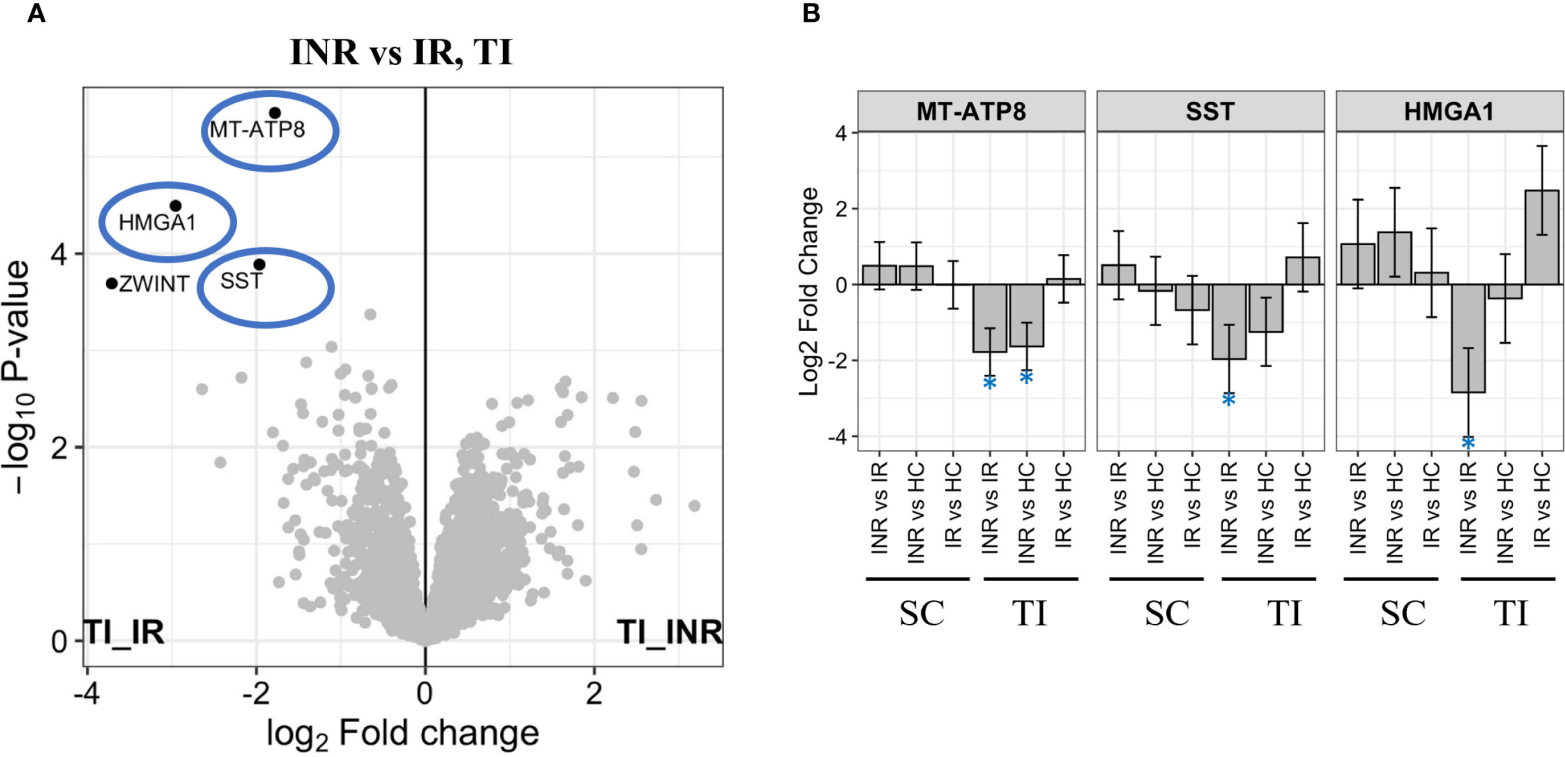
Protein regulation in terminal ileum **(A)** Volcano plot illustrating proteins most significantly downregulated in INR versus IR in terminal ileum. Y-axis shows -log_10_ adjusted p-value, X-axis log_2_ fold change. Significant DEPs showed in black dots with the corresponding name. Grey dots show non-significant DEPs. The most significantly downregulated proteins in INR *vs* IR (-log_10_ adjusted p-value > 2) highlighted in blue circles; **(B)** Level of most significantly downregulated factors in INR versus IR, in all groups and tissues. Y-axis shows log_2_ fold change. Error bars represent 95% confidence intervals. INR, immunological non-responders; IR, immunological responders; HC, healthy controls; DEPs, Differential Enrichment analysis of Proteomics data; HMGA1, *High mobility group AT-hook 1*; MT-ATP8, *Mitochondrially encoded ATP synthase membrane subunit 8*; SC, sigmoid colon; SST, *Somastatin*; TI, terminal ileum.

In terminal ileum, the *Mitochondrially encoded ATP synthase membrane subunit 8* (MT-ATP8), *High mobility group AT-Hook 1* (HMGA1), and the growth hormone release-inhibiting factor *Somatostatin* (SST) were all significantly downregulated factors in INR compared to IR (**Figures 7A, B**). MT-ATP8 (57, 58) and HMGA1 (59) have been implicated in mitochondrial function. In addition, the mitotic check point signaling factor *ZW10 interacting kinetochore protein* (ZWINT) was downregulated in INR compared to IR in ileum.

#### 3.2.3 Integrated transcriptomic and proteomic profiling reveals candidate biomarkers and therapeutic targets in INR

**Figure 8:**
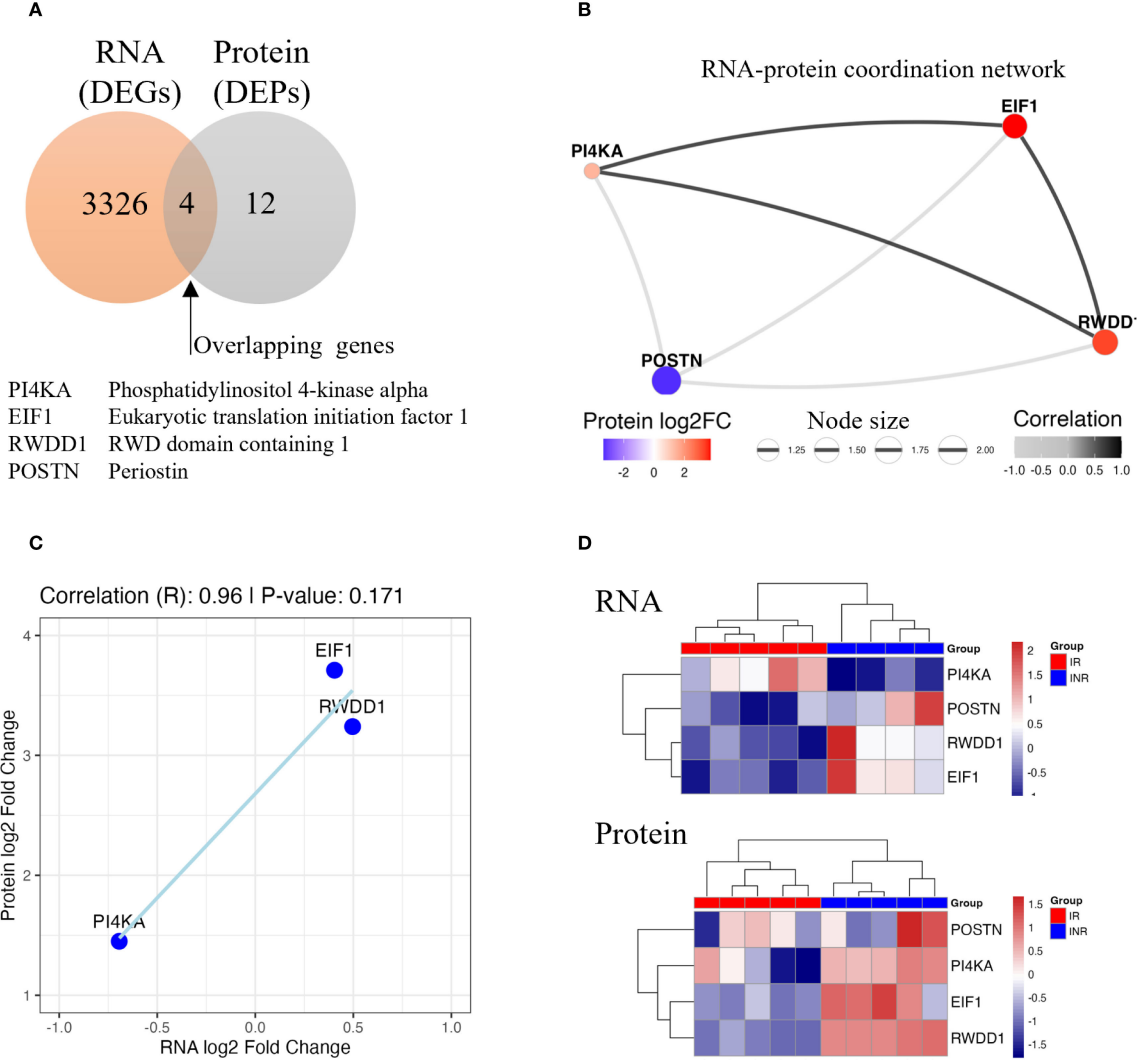
Integrated analysis of RNA-seq and proteomic data in colon samples from INR compared to IR. **(A)** Venn diagram showing the overlap between DEGs and DEPs. B) RNA–protein coordination network of overlapping genes. Node size reflects the product of absolute RNA and protein log_2_ fold changes; edge thickness represents correlation strength. **(C)** Scatterplot showing the positive correlation trend in three overlapping genes (EIF1, RWDD1, and PI4KA). **(D)** Heatmap of RNA and protein expression levels for overlapping genes. Samples clustered distinctly by group, separating INR from IR. INR, immunological non-responders; IR, immunological responders; DEG, differentially expressed gene; DEP, differentially expressed protein; log_2_FC, log_2_ fold change; RNA-seq, RNA sequencing.

To investigate the molecular mechanisms underlying discordant immunological response and to identify potential biomarkers, we conducted an integrated analysis of RNA-seq and proteomics data from gut mucosal biopsies of INR and IR. Among the 3,326 DEGs and 12 DEPs identified in INR versus IR in the colon, four genes showed concordant dysregulation at both transcript and protein levels (**Figure 8A**). These genes were PI4KA, EIF1, RWDD1, and POSTN. A weighted gene–protein correlation network revealed strong RNA–protein associations for these overlapping genes (**Figure 8B**). Specifically, PI4KA, EIF1, and RWDD1 were upregulated at the protein level, while POSTN was slightly downregulated. Discrepancies between mRNA and protein expression were observed for P14KA and POSTN. POSTN exhibited increased mRNA expression but was downregulated at the protein level in INR, suggesting the presence of post-transcriptional inhibitory mechanisms (60). In contrast, PI4KA was significantly downregulated at the mRNA level but upregulated at the protein level in INR compared to IR, indicating potential post-transcriptional upregulation or increased protein stability (60, 61) However, scatterplot analysis confirmed a strong positive correlation between RNA and protein expression for EIF1, RWDD1, and PI4KA, with a correlation coefficient of R = 0.96 (**Figure 8C**), indicating high concordance despite the limited sample size. Finally, unsupervised clustering of the integrated transcriptomic and proteomic data clearly distinguished INR from IR (**Figure 8D**), suggesting these four genes form a distinct molecular signature associated with impaired immunological response upon ART.

## 4 Discussion

This study provides the first integrated transcriptomic and proteomic analysis of mucosal tissues from people with HIV (PWH) who fail to achieve immune reconstitution (INR) despite effective ART, compared with immune responders (IR) and healthy controls (HC). We demonstrate that the sigmoid colon is a key site of molecular dysregulation in INR. INR exhibited broad transcriptional and proteomic alterations, including downregulation of immune and metabolic pathways, particularly those involving B cell responses, mitochondrial function, and RNA metabolism. These changes were not observed in the ileum and suggest a region-specific impairment in mucosal immune homeostasis in INR.

### 4.1 Regional differences emphasize the sigmoid colon as a key site of dysregulation

The most striking differences in gene and protein expression between INR and comparator groups (IR and HC) were found in the sigmoid colon. Transcriptomic analysis identified 3,326 differentially expressed genes (DEGs) between INR and IR in the colon, compared to no significant DEGs in the ileum. Similarly, proteomic analysis revealed 12 differentially expressed proteins (DEPs) in the colon and only four in the ileum. These findings underscore a compartmentalized pattern of immune dysfunction and highlight the distal colon as a uniquely affected site in INR.

The basis for this regional specificity is not fully understood. However, prior studies have reported that the colon may harbor higher levels of residual HIV replication and microbial translocation than the small intestine, which could drive ongoing mucosal immune activation and injury despite ART (3, 62). Our findings support this concept, revealing persistent molecular disturbances in colonic tissue from INR that are absent in the ileum.

### 4.2 Downregulation of immune, metabolic, and stress-response genes in colon of INR

The sigmoid colon of INR exhibited extensive transcriptional suppression, particularly affecting key immunological, metabolic, and stress-response pathways. Gene ontology and pathway enrichment analyses revealed significant downregulation of genes involved in protein transport, cytokine signaling, and adaptive immune responses, suggesting a compromised mucosal immune environment. Notably, CD4 mRNA expression was reduced by 37% in INR compared to IR.

We have previously reported that INR have approximately 10% fewer CD4+ T cells in the mucosa of the colon with increased activation and exhaustion of CD4+ T cells, correlating with signs of epithelial damage and mucosal dysfunction (16, 17). The magnitude of CD4 mRNA reduction observed here, suggests that, in addition to the reduced number of CD4+ T cells, INR also have transcriptional downregulation of the CD4 gene itself. This implies that impaired CD4 transcription, in combination with chronic immune activation (2, 3), may contribute to the failure of CD4+ T-cell recovery in INR, extending the mucosal dysfunction initially triggered by CD4^+^ T-cell loss in PWH (16, 17). The HIV Vpu protein and CD4 gene polymorphisms may further suppress CD4 expression (61, 62). Thus, reduced gut mucosal CD4 mRNA is not merely a consequence but also a contributing factor to poor immune reconstitution in INR (3, 28, 29).

Several key immune-regulatory genes were also downregulated in INR, including *TGFB1* and *IL21R*, which play crucial roles in immune modulation, tissue repair, and T/B cell function (33, 34). Similarly, downregulation of *HLA-E*, a molecule involved in NK cell regulation and epithelial immune tolerance, supports the hypothesis of impaired mucosal immune regulation (35, 36).

In contrast, a limited subset of genes associated with inflammation was upregulated. These include *CXCL1*, a chemokine known to enhance HIV replication (30, 32), *PTPN22*, which modulates TCR signaling and T cell activation (37), and *GZMA*, a granzyme involved in cytotoxic lymphocyte function mediating potential harmful effects on the host (38).

Additional dysregulated genes with recognized roles in HIV pathogenesis included MAP3K5, implicated in stress-induced apoptosis and interactions with HIV proteins (39), and CIITA (–1.138), a master regulator of MHC class II expression and antigen presentation (42). Dysregulation of CIITA further underscores impaired adaptive immune responses in INR, with direct implications for HIV infection.

Proteomic analysis confirmed transcriptomic findings and revealed increased expression of pro-apoptotic and stress-response proteins such as *CASP3* and *ACE2*, the known receptor for SARS-CoV-2 entry, both previously implicated in HIV-mediated epithelial damage and mucosal dysfunction (32, 56, 63). CASP3, a central mediator of apoptosis, was also elevated in INR. This aligns with previous evidence that HIV-1 envelope proteins can trigger CASP3 dependent apoptosis in CD4^+^ T cells through CD4 receptor engagement (32, 56). Additional upregulated proteins included *EIF1*, *RWDD1*, and *PI4KA*, associated with translational control and intracellular signaling (45, 47, 64). Conversely, *MSI2*, a key regulator of RNA metabolism and autophagy (65), and *POSTN*, essential for extracellular matrix remodeling and tissue repair (48), were significantly downregulated, suggesting impaired tissue homeostasis and repair capacity.

The differential expression of structural and mitochondrial proteins, such as *SPTBN1* and *TIMM8A*, further supports the presence of cellular stress and compensatory remodeling in INR mucosa (54, 55). Together, these findings reflect widespread disruption of mucosal immune regulation, apoptosis control, antigen processing, and cellular maintenance in INR, despite ART-mediated viral suppression.

### 4.3 Reduced B cell function in the colonic mucosa of INR

B cell–related signaling emerged as one of the most consistently downregulated pathways in colon of INR compared to IR. Genes involved in antigen binding, the immunoglobulin production, and B cell activation were significantly suppressed. This included multiple immunoglobulin variable genes and key cytokine receptors.

Interestingly, immunoglobulin gene expression in INR was not significantly different from healthy controls, suggesting that the defect is not due to baseline normalization but rather to a failure of ART-mediated upregulation. This aligns with prior reports of deficient B cell responses in INR (33, 34) and indicates underlying dysfunction in mucosal B cell differentiation.

RNA deconvolution analysis revealed a reduction in plasmablast frequencies in INR, while memory B cell levels remained comparable. This suggests a specific impairment in B cell activation processes, particularly in proliferation and differentiation into plasmablasts during the immune response to HIV at the mucosal level.

### 4.4 Upregulation of inflammatory immune signatures in INR

Despite suppression of adaptive immune pathways, the colonic mucosa of INR exhibited increased expression of genes linked to inflammation, including NK cell–associated markers and epithelial stress mediators. REG3α, a known marker of epithelial inflammation, was significantly upregulated in the INR colon compared to HC in our dataset. We have previously reported elevated levels of REG3α in INR (16), and its persistent increase here likely serum reflects ongoing epithelial stress and persistent inflammation as a potential important phenotype in INR in the absence of effective adaptive immunity.

### 4.5 Proteomic evidence supports immune and metabolic imbalance

Proteomic profiling supported our transcriptomic findings, revealing dysregulation of proteins involved in translation, apoptosis, and mitochondrial function. The upregulated proteins EIF1, RWDD1, and PI4KA play roles in translation initiation and phosphoinositide signaling (45, 47, 64), and may represent stress-adaptive mechanisms.

In contrast, POSTN, involved in extracellular matrix remodeling and hematopoietic regulation, was downregulated, suggesting impaired tissue repair in INR (50, 66). Decreased expression of the immunoglobulin variable gene IGKV18 suggests impaired local antibody production. Increased levels of CASP3 and ACE2 further point to increased epithelial susceptibility and apoptotic signaling (32, 56). Indeed, in addition to being the receptor for SARS-CoV-2 entry, ACE2 plays a crucial role in modulating gut inflammation and gut microbiome and has also been implicated in the pathogenesis of simian immunodeficiency virus infection (63). Additional upregulation of mitochondrial and structural proteins (SPTBN1, TIMM8A) may reflect epithelial adaptation to sustained cellular stress (54, 55).

### 4.6 Limited transcriptomic and proteomic changes in the ileum point to localized dysfunction

In contrast to the colon, the terminal ileum exhibited minimal molecular disturbance, with no significant differentially expressed genes. At the proteomic level, lower levels of MT-ATP8, HMGA1, SST, and ZWINT, proteins involved in mitochondrial function and cellular proliferation, were observed. These findings support a localized rather than systemic mucosal dysfunction in INR, focused on the colon.

### 4.7 Concordant transcriptomic and proteomic markers of INR mucosal dysfunction

Integration of transcriptomic and proteomic datasets highlighted a small group of altered markers, EIF1, RWDD1, PI4KA, and POSTN, that distinguished INR from IR and HC. The observed discordance between mRNA and protein levels for PI4KA and POSTN highlights the complexity of gene regulation and suggests that post-transcriptional mechanisms are involved. PI4KA upregulation at the protein level despite transcript downregulation suggests enhanced translation efficiency or reduced degradation, which may influence phosphoinositide signaling, vesicle trafficking, or viral persistence (61). Although POSTN mRNA levels were elevated in INR colon tissue, its protein levels were suppressed, suggesting a role for post-transcriptional regulation (60). N6-Methyladenosine (m6A) methylation of POSTN mRNA, particularly in the 3′ UTR, could mediate enhanced mRNA decay or impaired translation (60). These molecules may serve as candidate biomarkers for persistent mucosal immune dysregulation and provide mechanistic insights into failed immune reconstitution.

### 4.8 Parallels and distinctions with IBD

Interestingly, these findings exhibit several features that might relate to Inflammatory Bowel Disease (IBD), particularly through shared mechanisms of immune dysregulation and impaired gut barrier function. Both B cell dysfunction and altered immune responses are hallmark features of IBD, characterized by dysregulated innate and adaptive immune responses [reviewed in (67)]. Moreover, the hypothesis that impaired mucosal barrier function contributes to incomplete immune recovery in INR aligns with the pathogenic mechanisms observed in IBD, where increased intestinal permeability can lead to translocation of luminal antigens and microorganisms, contributing to intestinal inflammation [reviewed in (68)].

Notably, a recent study has shown that REG3α expression is absent in normal colonic tissue but is significantly induced in inflamed colonic biopsies, suggesting its potential role as a marker of inflammation in colonic conditions (69). Immunohistochemistry analyses indicated that REG3α was absent in colon from healthy controls but markedly upregulated in inflamed colonic tissue, suggesting its potential as a biomarker for colonic inflammation (69). We observed increased inflammation and immune activation in INR (16), which are common features in IBD, marked by chronic intestinal inflammation that triggers immune responses through similar pathways (70). Additionally, dysregulation in the adaptive immune response, cytokine signaling, and cellular processes related to tissue repair and growth are observed in IBD, where immune signalling and metabolic pathways are frequently altered, resulting in tissue damage and impaired repair mechanisms (67).

Furthermore, dysregulation of the adaptive immune response, cytokine signaling, and cellular mechanisms associated with tissue repair and growth has been noted in IBD (67). Within this framework, immune signaling and metabolic pathways frequently undergo alterations, resulting in tissue damage and hindered repair processes (66, 69).

While similarities can be identified between INR and IBD concerning disrupted immune and repair pathways, it is essential to acknowledge that persistent immune activation is also evident in IR, representing a shared characteristic among people living with HIV (70, 71). Consequently, instead of implying a direct disease overlap, upcoming research should investigate whether specific pathways or markers identified in IBD may have relevance for HIV-associated immune dysregulation, including INR (68, 70).

It is also important to emphasize that gut mucosal dysfunction should not be considered the sole driver of inflammation in PWH. Other contributing factors—including coinfections, immune senescence, and residual HIV viral replication—play significant roles in sustaining chronic immune activation (13, 15, 16).

Given these shared features, future research may benefit from exploring potential overlaps in pathways and biomarkers between IBD and INR, which could reveal therapeutic targets applicable to both. Specifically, dysregulation in B cell-mediated immunity, NK-cell interactions, and immune signaling pathways involved in tissue repair present viable avenues for targeted therapies. However, while such overlaps offer promising leads, these strategies must be carefully tailored to the distinct immunological context of INR (29, 70). A deeper understanding of the molecular mechanisms driving immune dysregulation in INR is essential before translating IBD-derived treatments, with careful consideration of safety, efficacy, and potential interactions with HIV-specific management strategies.

### 4.9 Strengths, limitations, and future directions

The strength of our study is the well characterized study groups matched on ethnicity, age, and sex for all groups and nadir CD4 T^+^ count for INR and IR. The biopsies were collected in the same hospital by the same gastroenterologist, from the same parts of the gut, using a well-defined protocol avoiding anatomical bias. Another important aspect of this study is that we investigated both the transcriptomic and proteomic regulation, which provide new insight to the pathogenesis of INR and a resource for developing novel adjuvant therapy. This study also has limitations such as low number of individuals and limited sample material per individual. Only one biopsy per individual was analyzed for RNA or protein regulation. As biopsies only sample a limited area of the mucosa, this can result in sampling bias. Finally, whereas we found markedly different regulation of several transcripts and proteins between INR and IR, the molecular mechanisms for these findings are not clear and the observed associations may not reflect direct causal relationships. Thus, functional validation and mechanistic investigation of these findings, such as through *in vitro* HIV infection models, B cell co-culture systems, or organoid-based approaches, are necessary to establish direct mechanistic roles, and should be prioritized in future studies.

## 5 Conclusions

We have shown that INR display a highly altered mucosal transcriptome and proteome compared to IR in the sigmoid colon, but not in the terminal ileum. This underscores previous findings implicating sigmoid colon, as opposed to terminal ileum, as an anatomic site linked to incomplete immune recovery in PWH. Our findings suggest that the impaired immunological response in INR is associated with extensive transcriptional and protein dysregulation in the sigmoid colon.

The differentially regulated factors should be candidates for further research targeting adjuvant therapy to improve the prognosis and quality of life for PWH and incomplete immune recovery. Integration of transcriptomic and proteomic datasets highlighted a small group of dysregulated markers, PI4KA, EIF1, RWDD1, and POSTN, that distinguished INR from IR and HC. These molecules may serve as candidate biomarkers for persistent mucosal immune dysregulation and provide mechanistic insights into failed immune reconstitution. The shared mechanisms of immune dysregulation and impaired gut barrier function observed in both INR and IBD suggest a promising avenue for exploring common therapeutic targets between these conditions. However, given the distinct immunological challenges posed by INR, further research is essential to fully understand the molecular underpinnings of these dysregulations and to adapt IBD treatment strategies accordingly.

## Data Availability

The datasets presented in this study can be found in online repositories. The names of the repository/repositories and accession number(s) can be found in the article/**Supplementary Material**.
