# Supplementary figures and images for: Transcriptomic and proteomic profiling reveal immune and metabolic dysregulation in the colonic mucosa of people living with HIV with incomplete immune recovery
Source: Front Immunol. 2025 Sep 17;16:1635523. doi: 10.3389/fimmu.2025.1635523 (PMC12484174; doi:10.3389/fimmu.2025.1635523)

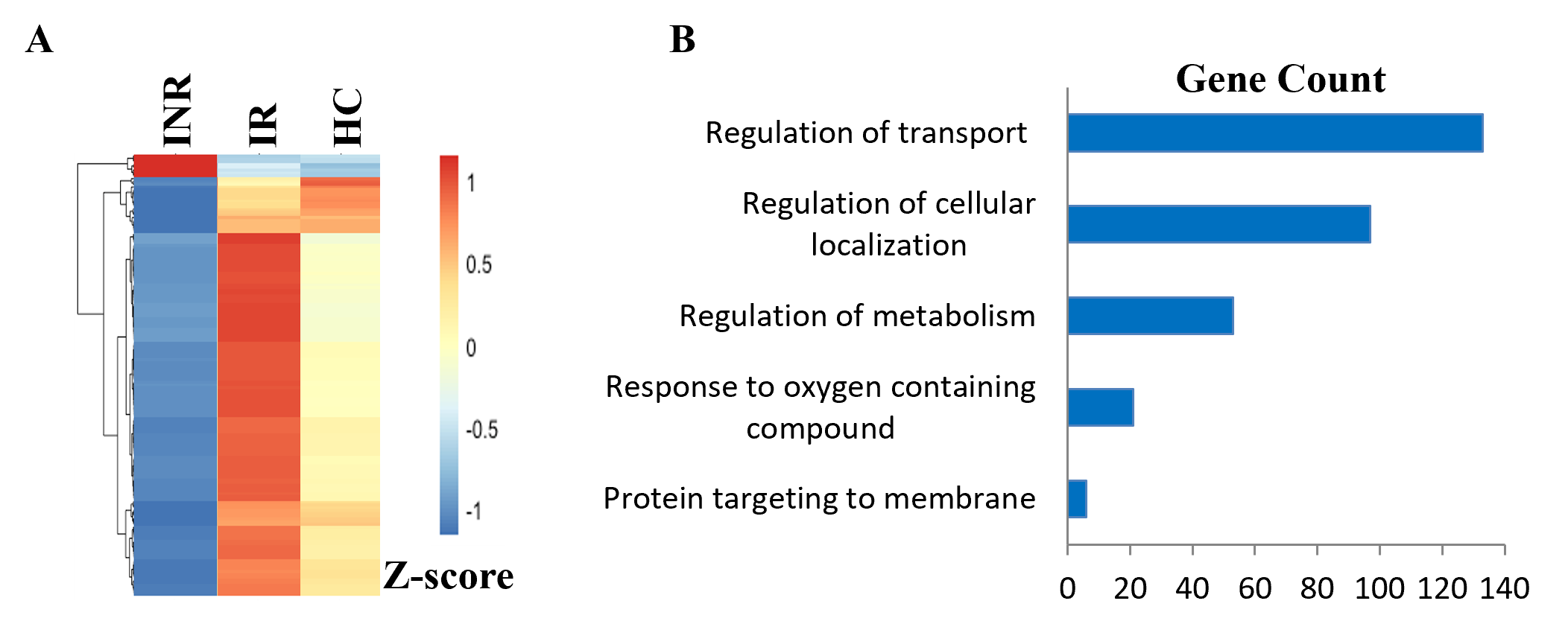

Supplement: Supplementary file 1 [file Image1.tif]

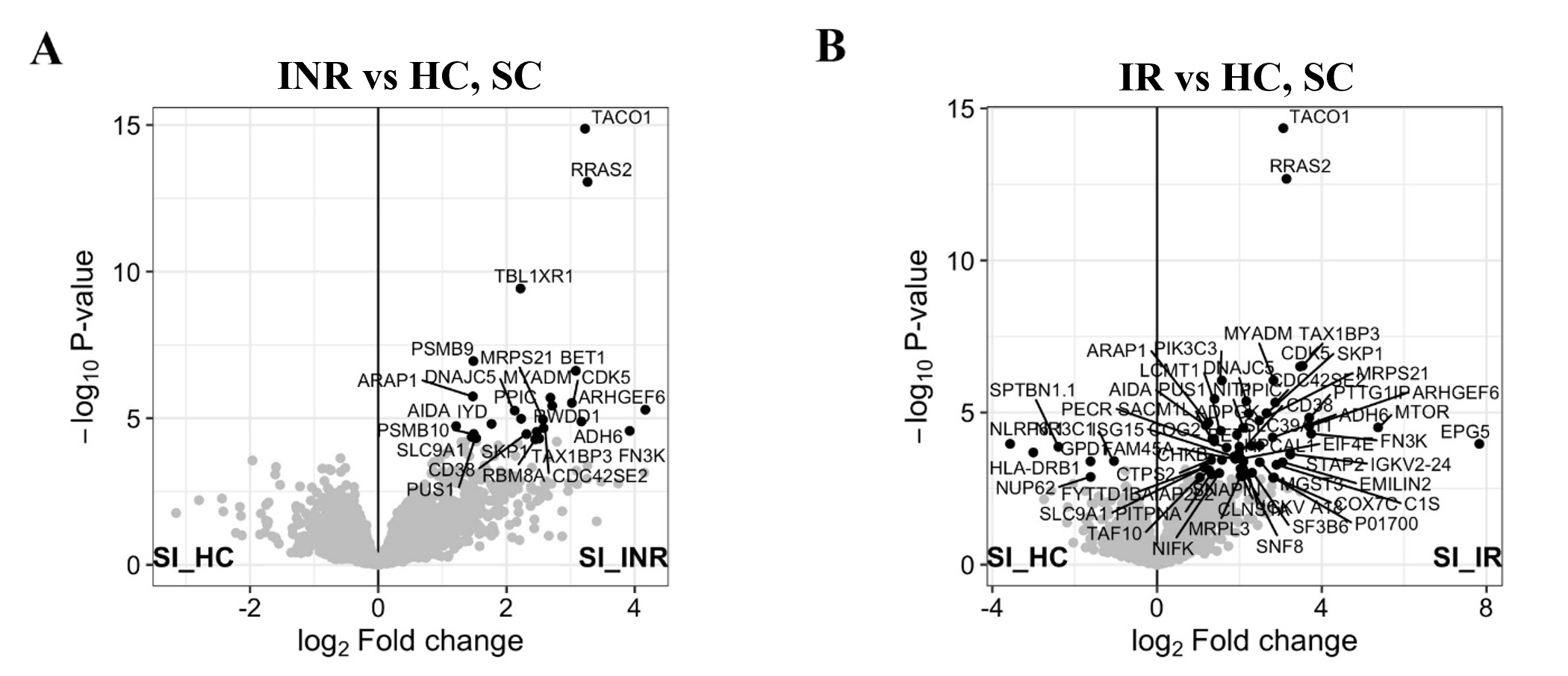

Supplement: Supplementary file 2 [file Image2.tif]

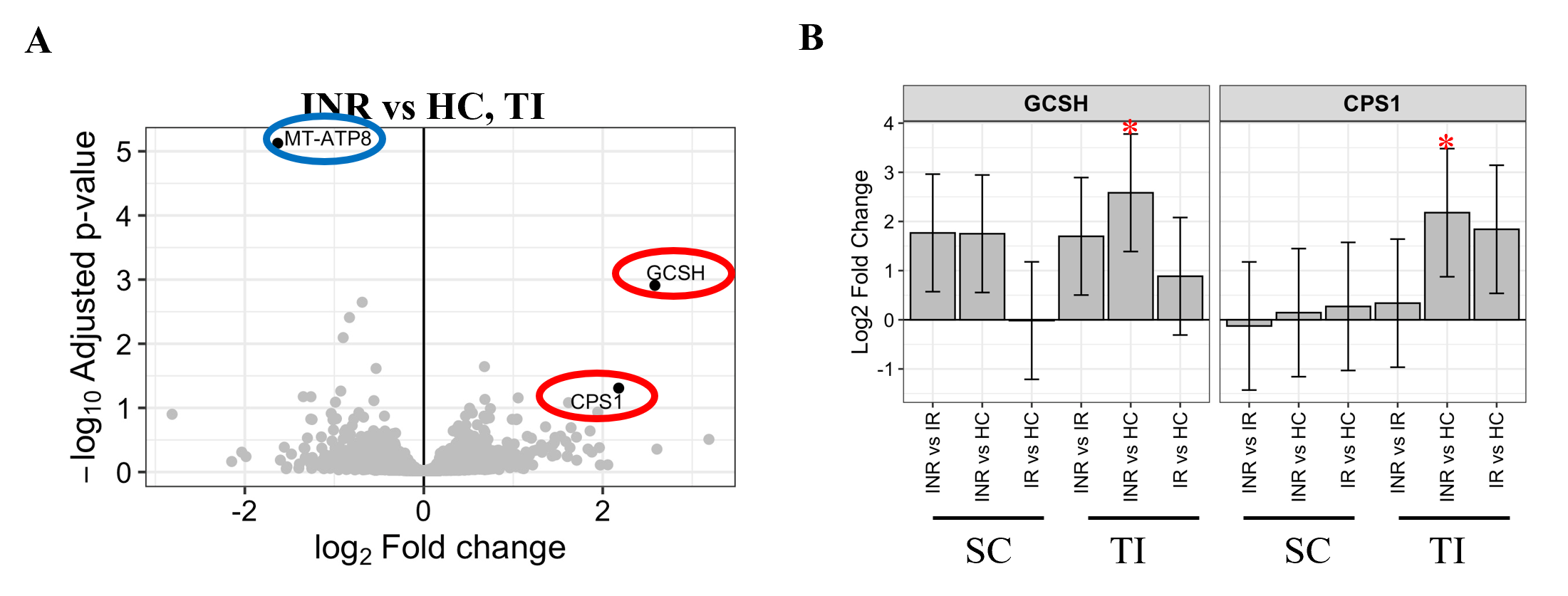

Supplement: Supplementary file 3 [file Image3.tif]
