# Supplementary material for: Transcriptomic and proteomic profiling reveal immune and metabolic dysregulation in the colonic mucosa of people living with HIV with incomplete immune recovery
Source: Front Immunol. 2025 Sep 17;16:1635523. doi: 10.3389/fimmu.2025.1635523 (PMC12484174; doi:10.3389/fimmu.2025.1635523)
Supplement: Supplementary file 6 [file Table3.docx]

**Supplementary Material and Methods**

**RNA-sequencing (RNA-Seq)**

Reads containing adapter sequences and low-quality reads were trimmed/removed using BBDuk (part of BBMap v34.56; parameters: ktrim=r k=23 mink=11 hdist=1 tbo tpe qtrim=r trimq=15 maq=15 minlen=36 forcetrimright=149) (1). Clean reads in FASTQ format were aligned against the ENSEMBL Human GRCh38 release 90 genome using TopHat v2.0.13 (Bowtie 2.2.3.0) (parmaters: --library-type fr-firststrand --no-mixed --no-novel-juncs) (2). Reads mapping to genes were counted using featureCounts v1.4.6-p1 (parameter: -p -s 2) (3). Differentially expressed (DE) genes from RNA-seq data were calculated using DESeq2 v1.18.1 in R v3.4.2 (4). DEGs were processed and visualized using R packages, including ggplot2, tidyverse and pheatmap (V1.0.12).  Pathway and process enrichment of Gene Ontology Biological Processes analyses (GO) were performed using the R package clusterProfile (version 3.18.1) using the default settings. The list of DEGs for all comparisons, including expression levels, p-value and adjusted p-value are included in Supplementary Table 1.

**Liquid Chromatography–Tandem Mass Spectrometry (LC-MS/MS)**

Prior to LC-MS/MS, the protein pellets were resuspended in 8 M urea, 30 mM Tris pH 8.0, 4 % CHAPS containing 5 mM TCEP and incubated for 1 hour at room temperature with shaking. Twenty µg protein were alkylated with iodoacetamide (1 µmol/mg protein) for 30 min in the dark, followed by precipitation using methanol-chloroform and overnight trypsin (Thermo Fisher Scientific, Waltham, MA) digestion in 50 mM NH_4_HCO_3_ at 1:50 ratio (w/w, enzyme:protein) at 37^◦^C with shaking (5). Samples were desalted using in-house C18 stage-tip columns, dried and resuspended in 0.1 % formic acid prior to centrifugation at maximum speed (16 000 g) to remove insoluble particles. Protein analyses were performed on an LC-MS/MS platform consisting of an EASY-nLC 1200 UHPLC system coupled to a Q Exactive HF mass spectrometer operating in FullMS-ddMS2 mode (Thermo Fisher Scientific, Waltham, MA). Peptides (2 µg) were injected onto an Acclaim PepMap C18 column (75 µm i.d. × 2 cm nanoviper, 3 µm particle size, 100 Å pore size) (Thermo Fisher Scientific, Waltham, MA) and further separated on an EASY Spray^TM^ LC column (75 µm i.d. × 50 cm nanoviper, 2 µm particle size, 100 Å pore size) (Thermo Fisher Scientific, Waltham, MA) at 40^o^C. The following 180 min method was used at 250 nl/min flow rate: starting with 2 % solvent B (100 % Acetonitrile, 0.1% Formic acid) with an increase to 40 % solvent B for 150 min, followed by an increase to 100 % solvent B over 20 min, and held for 10 min. Solvent A consisted of 0.1% Formic acid. The peptides eluting from the column were ionized by an Easy Spray^TM^ Source (Thermo Fisher Scientific, Waltham, MA) and analysed on the mass spectrometer operating in positive-ion mode using a spray voltage of 1.75 kV and HCD fragmentation with normalized collision energy (NCE) 28. Each MS scan (300 – 1800 m/z) was acquired at a resolution of 120 000 FWHM, automatic gain control (AGC) target value of 3 × 10^6^, maximum injection time (mIT) of 100 ms, followed by 15 MS/MS scans acquired at a resolution of 60 000 FWHM, AGC of 1 × 10^5^ with a maximum injection time (mIT) of 100 ms and isolation window 1.2 m/z. Proteins were quantified by processing MS data using MaxQuant (MQ) v.1.6.6.0 (6).  The preview version 2.3.5 from Protein Metrics Incorporate (7) was used to inspect the raw files to determine optimal search criteria. Namely, the following search parameters were used: enzyme specified as trypsin with a maximum of two missed cleavages allowed; acetylation of protein N-terminal, oxidation of methionine, and deamidation of asparagine/glutamine as dynamic post-translational modifications. These were imported in MQ which uses mass-charge and retention-time values to align each run against each other sample with a minute window match-between-run function and 20 mins overall sliding window using a clustering-based technique. These were further queried against the Human proteome including isoforms downloaded from Uniprot (<https://www.uniprot.org/proteomes/UP000005640>) in June 2019 along with MQ’s internal contaminants database using Andromeda built into MQ. MS/MS tolerance was set to 20 ppm with both protein and peptide identifications false discovery rate set to 1%, so that only high-confidence unique peptides were used for final protein group identification. Peak abundances were extracted by integrating the area under the peak curve. Each protein group abundance was stabilized and normalized to the total abundance of all identified peptides, using the calculated median of summed unique and razor peptide abundances for each protein, using label-free quantification (algorithm (8)) with minimum peptides ≥ 1. Raw data exported from MaxQuant were further analysed mainly using an R DEP package (Differential Enrichment analysis of Proteomics data, Version 1.12.0). In brief, the data were cleaned by removing false hits, including contaminants, reverse proteins, and proteins identified by site. The proteins with label-free quantification value (LFQ.intensity) at zero in all replicates in at least one condition were filtered. The data were normalized by variance stabilizing transformation (vsn), a limma based model. Then, the missing values were imputed by MinProb, a method suitable for missing-not-at-random (MNAR) data, which randomly draws minimal values from a left-shifted distribution. The differentially regulated proteins were identified using the protein-wise linear model (limma package inside DEP) combined with empirical Bayes statistics. DEPs are defined by p.adj (alpha) at 0.05 and minimum log fold change of 2. Gene ontology (GO), KEGG pathway and Reactome enrichment analysis were performed using R package clusterProfile (3.18.1) and ReactomePA (1.34.0). The data were visualized using R packages including ggplot2 and VennDetail. Data are available via ProteomeXchange with identifier PXD041255.

Reference List

1. Bushnell B. BBTools. Available from: <https://sourceforge.net/projects/bbmap/>. 2014.

2. Kim D, Pertea G, Trapnell C, Pimentel H, Kelley R, Salzberg SL. TopHat2: accurate alignment of transcriptomes in the presence of insertions, deletions and gene fusions. Genome biology. 2013;14(4):R36.

3. Liao Y, Smyth GK, Shi W. featureCounts: an efficient general purpose program for assigning sequence reads to genomic features. Bioinformatics. 2014;30(7):923-30.

4. Love MI, Huber W, Anders S. Moderated estimation of fold change and dispersion for RNA-seq data with DESeq2. Genome biology. 2014;15(12):550.

5. Wessel D, Flügge UI. A method for the quantitative recovery of protein in dilute solution in the presence of detergents and lipids. Analytical biochemistry. 1984;138(1):141-3.

6. Tyanova S, Temu T, Cox J. The MaxQuant computational platform for mass spectrometry-based shotgun proteomics. Nature protocols. 2016;11(12):2301-19.

7. Kil YJ, Becker C, Sandoval W, Goldberg D, Bern M. Preview: a program for surveying shotgun proteomics tandem mass spectrometry data. Analytical chemistry. 2011;83(13):5259-67.

8. Cox J, Hein MY, Luber CA, Paron I, Nagaraj N, Mann M. Accurate Proteome-wide Label-free Quantification by Delayed Normalization and Maximal Peptide Ratio Extraction, Termed MaxLFQ*. Molecular & Cellular Proteomics. 2014;13(9):2513-26.
